# Embodying the camera: An EEG study on the effect of camera movements on film spectators´ sensorimotor cortex activation

**DOI:** 10.1371/journal.pone.0211026

**Published:** 2019-03-13

**Authors:** Katrin Heimann, Sebo Uithol, Marta Calbi, Maria Alessandra Umiltà, Michele Guerra, Joerg Fingerhut, Vittorio Gallese

**Affiliations:** 1 Department of Medicine and Surgery, Unit of Neuroscience, University of Parma, Parma, Italy; 2 Department of Food and Drug, University of Parma, Parma, Italy; 3 Department of Humanities, Social Sciences, and Cultural Industries, University of Parma, Parma, Italy; 4 Berlin School of Mind and Brain, Humboldt-Universität zu Berlin, Berlin, Germany; 5 Institute of Philosophy, School of Advanced Study, University of London, London, United Kingdom; University of Bologna, ITALY

## Abstract

One key feature of film consists in its power to bodily engage the viewer. Previous research has suggested lens and camera movements to be among the most effective stylistic devices involved in such engagement. In an EEG experiment we assessed the role of such movements in modulating specific spectators´ neural and experiential responses, likely reflecting such engagement. We produced short video clips of an empty room with a still, a zooming and a moving camera (steadicam) that might simulate the movement of an observer in different ways. We found an event related desynchronization of the beta components of the rolandic mu rhythm that was stronger for the clips produced with steadicam than for those produced with a still or zooming camera. No equivalent modulation in the attention related occipital areas was found, thus confirming the sensorimotor nature of spectators´ neural responses to the film clips. The present study provides the first empirical evidence that filmic means such as camera movements alone can modulate spectators’ bodily engagement with film.

## Introduction

In the last decades, the perspective of embodied cognition in the cognitive sciences has become increasingly influential. Within this development, it has been suggested that not only our everyday perceptual and cognitive tasks, but also the experience of cultural artifacts and works of art, like paintings and movies, is mediated by bodily resonance processes and by motor system responses to physical and medial qualities of these objects [1, 2, 3, 4, 5]. A neuronal mechanism that has been suggested to play a major role in this phenomenon is the so-called mirror mechanism. This term commonly refers to an action-perception link—originally discovered in macaque monkeys—in which a class of neurons within the premotor cortex have been found to respond not only to the execution of goal-related actions but also to the observation of similar actions when executed by others [6]. The existence of such action-perception link has by now also been firmly supported in humans [7, 8, 9, 10, 11], and a vast range of research has addressed its possible function within a variety of cognitive tasks such as action understanding [11,12], empathy [13] as well as aesthetic experience [1, 14].

The present study investigates the perceptual experience of film and the neural mechanisms involved. Film is one of the most important media of our times, used extensively for purposes of information and education as well as for advertisement and amusement. One of the key features of its success consists in its capacity to strongly and bodily engage the public. While watching a feature film, we laugh, weep, jump, get angry at or feel with the characters acting on screen. This power of engagement is integral part of spectators’ filmic experience.

The present research focuses on camera usage as one of the central narrative devices used by film makers. One can separate two ways of creating movement by means of the camera: 1) lens movements such as zoom, related to the changing of the field of view; and 2) physical movement of the camera, which, in turn, can be differently accomplished, for example by moving a hand-held camera, by moving it along tracks, also known as dolly, or by securing it to the body of a moving camera man and slightly correcting for disturbances: the so-called steadicam. According to film theory and film-makers, the usage of such means is central for spectators’ experiences, particularly so for their feeling of realism and immersion in the film scenes [15, 16]. While a still camera can provide a strong impression of realism by completely hiding the act of active filming and thereby chosen perspective, skilled lens and camera movements are able to boost our immersion in the film by adding kinesthetic bodily cues as well as the sense of balance and gravity. As noted by film theorist David Bordwell [15], camera movements are indeed the basis for the orthodox comparison between the camera and the human body. The head may rotate, i.e., pan or tilt; the entire organism may displace itself, i.e., by tracking. Lens and camera movements therefore are a “persuasive surrogate for our subjective movement through an objective space” [15] (p. 23).

Empirical research recently has picked up on this, taking into consideration the importance of the body and its habits in shaping the film space. Specifically, it investigated the role of filmic stylistic means in enabling spectators’ bodily engagement [16, 2, 3, 4, 17, 18, 19, 5].

In a previous study, we showed that different usages of the camera when filming a goal-directed action have a modulating effect on the mirror mechanism of spectators´ watching the respective film clips [19]. A particularly strong modulation was reported for the steadicam that has previously also been described as the means best simulating a movie character’s view and movement in the scene, thus fostering an enhanced involvement of the spectator [20].

To assess this hypothesis, we conducted a high-density EEG experiment, in combination with spectator interviews, focusing on spectators´ neural and experiential responses to film clips produced by different uses of the camera. Specifically, we focused on sensorimotor cortex activation by measuring Event Related Desynchronization (ERD) of the mu rhythm, a standard marker of “motor resonance” [21, 22, 23, 24, 25, 26], during action observation. To be precise: previous research showed that power in the frequency band components of this rhythm decreases during sensorimotor activation. Since observation of scenes portraying human actions as used in the study referred to above [19], may by itself generate spectators’ motor resonance to those actions, by activation of the mirror mechanism, we decided to film an empty room devoid of any acting agent. The hypothesis underlying this study was therefore that we might engage with the camera (and the succession of frames produced by the camera) by showing differential sensorimotor activation even when there is no depicted action visible. We filmed the same room in three different ways: a) by means of a still camera mounted on a tripod (control condition of similar visual input without camera/lens movement); b) by zooming in on the room (lens movement); c) by using a steadicam allowing moving through the room (camera movement). We hypothesized that all clips involving camera and possibly lens movements would elicit sensorimotor activity. More precisely, any clips involving camera/lens movements should lead to a typical ERD-ERS pattern in the frequency bands and location correlated to sensorimotor activity (that is, components of the Rolandic mu- rhythm including alpha and beta measured from central electrodes, see EEG recording and analysis section below). Moreover, the ERD in particular should be significantly stronger (that is, frequency power should be lower) for the camera and possibly lens movement conditions (Steadicam and Zoom) when compared to the Still condition (control without movement). Furthermore, we assumed that because the camera movement accomplished by using the steadicam is presumably the one that better approximates the movements of a moving observer than the zooming camera, images produced by the steadicam should also induce a stronger ERD than that a zoom.

## Materials and methods

### Participants

18 healthy volunteers, recruited by public announcement, participated in the experiment (5 males, 13 females, mean age 24.3 (Standard Deviation +/-4.3), all right handed as assessed by the Edinburgh Handedness Inventory [27]. Before the experiment, all participants received written and oral experimental instructions and gave their informed written consent. After the experiment, each participant was debriefed and received 25 Euro as reimbursement. The study was approved by the local ethical committee in Parma (Comitato Etico per Parma, Azienda Ospedaliero-Universitaria di Parma, Azienda Unità Sanitaria Locale di Parma, Università degli Studi di Parma) and was conducted according to the principles expressed in the Declaration of Helsinki.

### Stimuli

**Fig 1:**
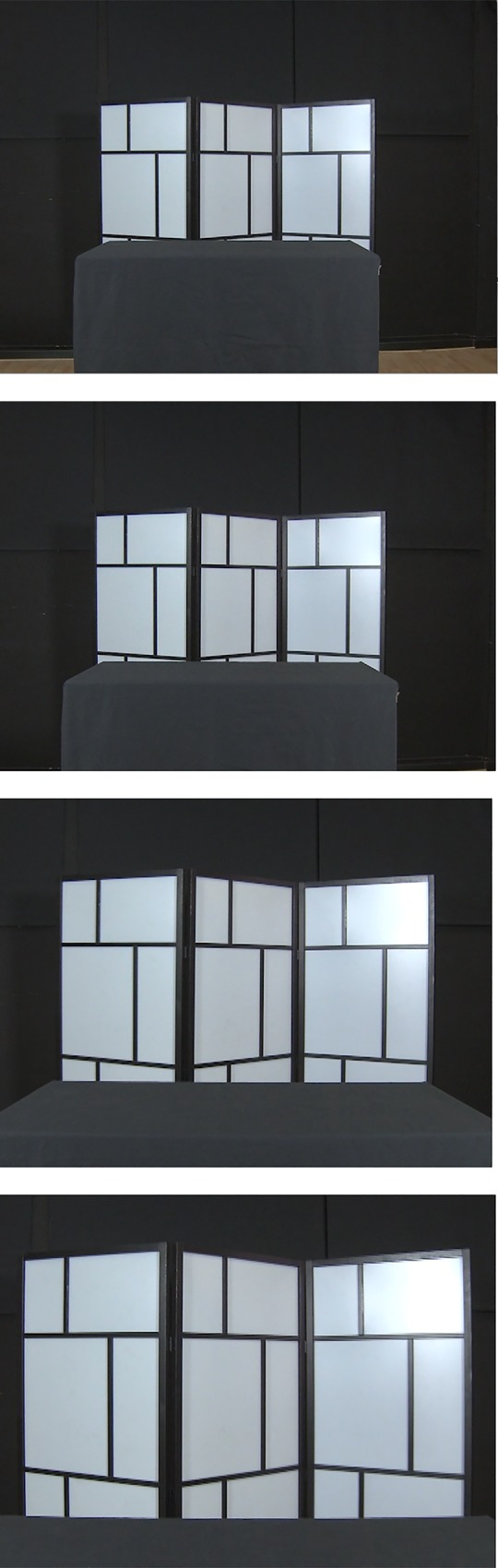
4 single frames extracted from Steadicam condition video clip: the camera started from a position, common to all conditions (first frame), and was consequently moved by a steadicam construction, carried by a professional camera man, to the end position (last frame). The two middle frames illustrate the picture change during this way.

Stimuli consisted of three different types of short (3000 ms) video clips showing an empty table in front of a panel with a black and white geometrical pattern to enhance the 3D perception of the room. The background wall was black. Video clips were recorded in a professional film studio, enabling us to film the same scene 7 times under highly controlled conditions, only varying the way the camera was used to record the scene. In one condition (guiding the production of 1 video), the camera was mounted on a tripod 230 cm from the table. We will refer to this condition as “Still condition”. In the second condition (guiding the production of 3 videos) we used an automatic zoom (predetermined in speed and start as well as end frame) to enlarge the scene (“Zoom condition”) and in the third condition (guiding the production of 3 videos) the camera was carried towards the table by a cameraman using a steadicam in such a way that the end frame matched that of the Zoom condition (“Steadicam condition”). In the supplemental material one video clip of each condition is provided for illustration. Fig 1 shows four still frames taken from a video clip filmed by steadicam including start and end positions to give a limited impression here.

### Experimental procedure

The experiment consisted of two parts: 1) a 60 minutes EEG recording session; 2) a 5 minutes interview period.

1) EEG

**Fig 2:**
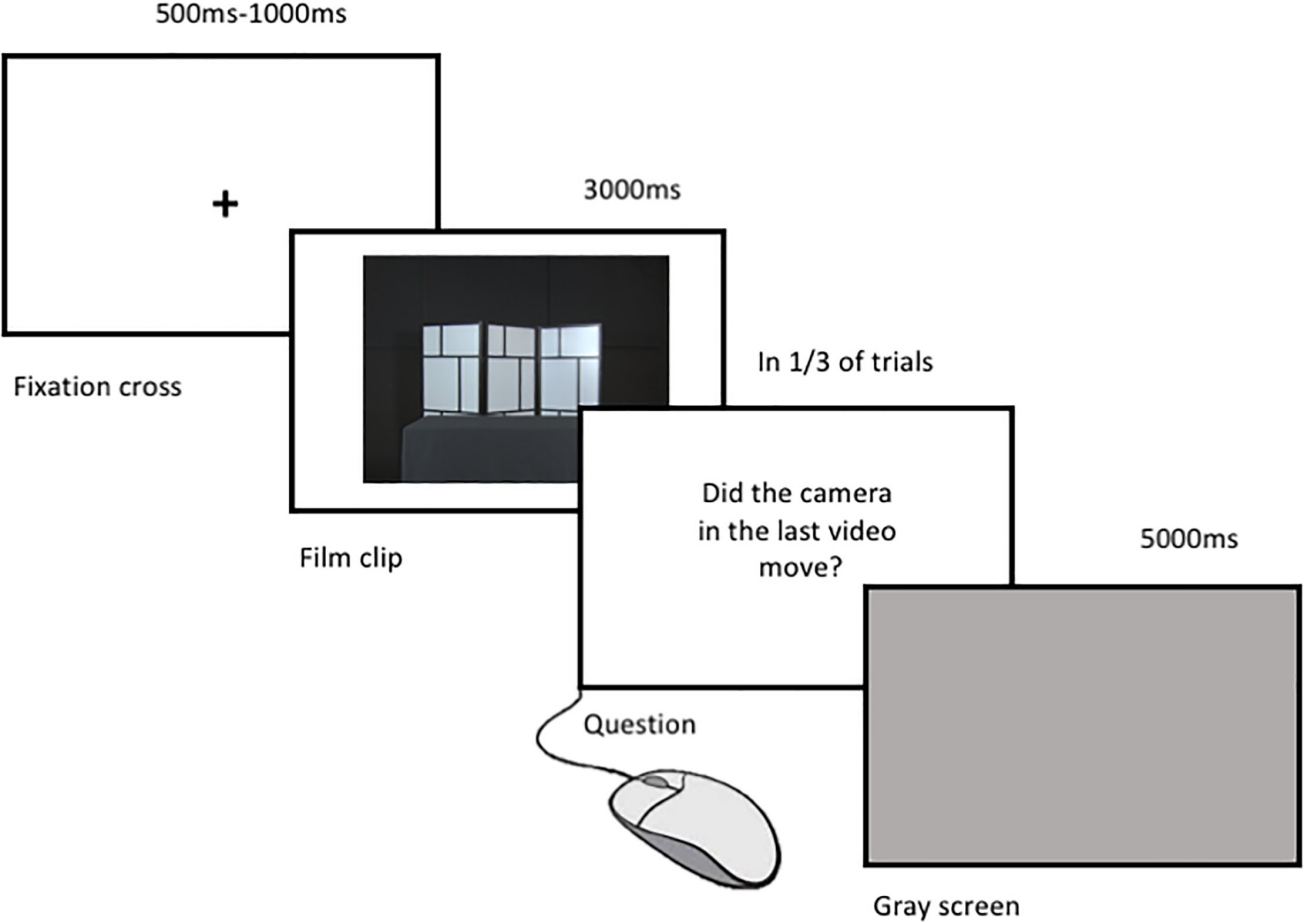
Experimental paradigm employed during EEG recording.

Participants were seated in a shielded EEG lab in front of a computer screen placed on a table at a distance of 50 cm. The EEG was recorded during four blocks, each separated by a short break. Each block consisted of 51–52 trials (in total 68–70 per condition: Still, Zoom, and Steadicam, for a total number of 207 trials). Each trial started with a fixation cross of 500–1000 ms (random length), followed by one out of the 7 videos of 3000 ms (counterbalanced across production conditions, see above). After stimulus presentation, a grey screen was displayed for 5 seconds allowing the signal to return to baseline. Participants were asked to blink only in the second half of the grey screen period to minimize motion artifacts. In one third of the trials, after stimulus presentation and before the grey screen, participants were asked a question regarding the last seen videos (e.g. “Did the camera in the last video move?”, answer possibilities yes/no, by pressing mouse buttons). This resulted in 69 of such action execution trials, randomly distributed in the whole sample. The answer had to be given by clicking one of the two buttons of the mouse positioned at 15 cm from participants´ right hand (“Action Execution condition”). If participants gave a wrong answer or did not answer within 3000 ms, they were informed that the answer was incorrect or given too late. This Action Execution condition served both as control for attention and to record participants’ ERD of the mu rhythm during right hand movements. For visualization of the trial protocol please see Fig 2.

During the entire recording, muscle-responses from hands and legs were measured using EMG, enabling the control for muscle movements in hand or feet that might confound results in the observation trials.

After the recording session, participants were shown the videos again (one for each condition: Still, Zoom, and Steadicam) and were asked whether they saw any differences between the videos and, in case they did, how they would describe them. We explicitly decided to use open questions rather than a rating task, to get some more detailed information about the spectators’ experiences.

### EEG and EMG recording and analysis

EEG data was acquired by a 128-channel Sensor Net (Electrical Geodesic, Eugene, USA) and recorded within standard EGI package Net Station 4.3.1. EEG was sampled at 500 Hz, electrodes impedance was kept below 50 KΩ (checked after each block). The raw EEG data was recorded with the vertex (Cz) as the online reference and re-referenced off-line to the common average [28]. Previous studies [29, 30] recommended the use of the EEG infinity referencing system (IR), also known as the reference electrode standardization technique (REST) [29]. Following such recommendation, as a second step, an approximate zero reference (REST) [31] was conducted by the rest_refer function from www.neuro.uestc.edu.cn/rest. Our results are reported using only the IR referencing system [32]. Stimuli were presented with E-Prime 2.0. All further processing was done using the Matlab toolbox FieldTrip [33]. EEG data were filtered off-line with a band-pass filter of 1–30 Hz and segmented into specific time epochs. From observation trials, the whole 3 sec of stimulus presentation plus the first second of grey screen (resynchronization phase) were analyzed. The following epoch of 1000 ms of gray screen (from 1000 to 2000 ms of the inter-trial interval) was taken as comparison period (baseline) for the later applied band selection per participant and the final statistics. From action execution trials, segments of 1000 ms were chosen starting 500 ms before the motor response (button press) and ending 500 ms after it.

In all trials, an EMG signal was recorded with a CED Micro 1401 (Cambridge Electronic Design, Cambridge, U.K.) connected to CED 1902 amplifier, and CED Spike (2) software was used for processing and inspection. Pairs of surface electrodes (Ag–AgCl, disposable, 7 mm × 4 mm) were attached to the 1^st^ dorsal interosseus muscle of both hands, and to the tibialis anterior muscle of the legs. Collected EMG signals were amplified (1000 ×), band-pass filtered (20–2000 Hz) and digitized at a sampling rate of 5 kHz. Spikes of 0.5 volt or more of peak amplitude were considered movement artifacts, leading to trial exclusion.

Further artifacts in the EEG (due to eye blinks, eye movements, pulse, sweating etc.) for all conditions were removed through a visually inspected Independent Component Analysis as implemented in FieldTrip, considering time, topographic and spectral distribution of the component, as well as consequent visual inspection and exclusion of all trials with still remaining artifacts. A minimum number of 45 trials for each condition was kept. Due to this condition, 2 out of 18 participants had to be excluded from further analysis.

To assess rolandic mu-rhythm modulation, a time–frequency analysis was performed on the data (all electrodes) of each participant on 1.5-second-long segments (adding 250 ms on start and finish of defined time periods to avoid artifacts to processing) for all conditions using Hanning tapers in 1 Hz intervals with a sliding time-window of 0.5 seconds in the frequency range from 7 to 30 Hz. Frequency-power coefficients were calculated by taking the average across trials. Data was corrected regarding pre-stimulus baseline (taken out of the fixation cross period) by division.

**Fig 3:**
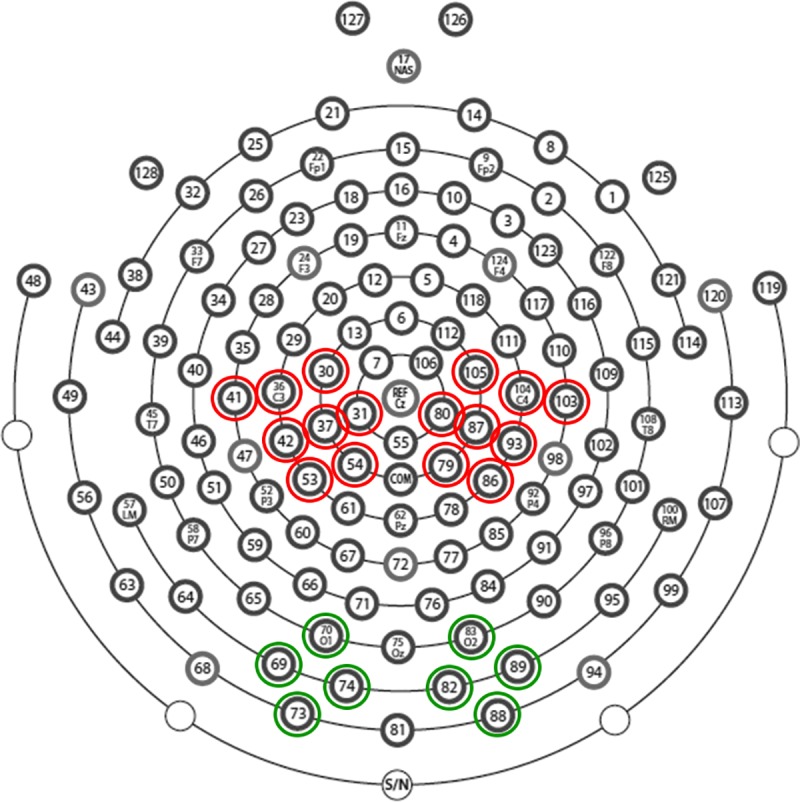
Hydrocel Geodesic Sensor Net– 128 channels map. Electrodes circled in red indicate central electrode-clusters of interest chosen to assess rolandic mu-rhythm modulation (electrodes 30, 31, 36, 37, 41,42, 53, 54 in left and 79, 80, 86, 87, 93, 103, 104, 105 in right). Electrodes circled in green indicate electrode-clusters of interest chosen to assess whether recordings in central areas might be affected by posterior activity (electrodes 69, 70, 73, 74 in left and electrodes 82, 83, 88, 89 in right).

Electrode-clusters of interest were chosen on the basis of previous studies: 8 electrodes in each hemisphere located around standard C3 and C4 sites (Electrodes 30, 31, 36, 37, 41,42, 53, 54 left and 79, 80, 86, 87, 93, 103, 104, 105 right; see 19, 30, 31, 32, 28, 33). Please see Fig 3 for location of the clusters.

Using these clusters and following the procedure described in previous studies [34, 35, 36], for each participant specific alpha-frequency bands were selected in the range of 8–14 Hz: the individual peak (F) of attenuated frequency was determined by calculating the ratio between the frequency power in action execution trials and during comparison period of grey screen observation (see above) in the six following sub-frequency bands: 8–9, 9–10, 10–11, 11–12, 12–13, 13–14 Hz. Each value was then transformed into a log-ratio and the frequency that corresponded to the log-ratio with the most negative value was taken as F. A 3 Hz range frequency band was chosen for each participant (F—1; F + 1). For the following statistical analyses, the individual frequency power in this 3 Hz range was extracted in all conditions. Since the central alpha frequency band (8–14 Hz) overlaps with the posterior alpha band, it is possible that recordings in central areas might be affected by this posterior activity. In order to control for this, for the alpha-range selected for each participant we performed an additional analysis in 4 electrodes per hemisphere in occipital areas (electrodes 69, 70, 73, 74 in left occipital lobe & electrodes 82, 83, 88, 89 in right occipital lobe) using the same frequency bands as previously described (see Fig 3).

Furthermore, in every participant frequency power values for two beta-frequency ranges were extracted (a lower band of 14–20 Hz and a middle band of 18–24 Hz) using the same central electrode-cluster as for the alpha-range (regarding range selection to collect beta component of the rolandic mu-rhythm, see [37] and results of [19]). Also for these ranges we controlled for effects in occipital areas to exclude the possibility of attention effects.

### Statistical analysis

#### EEG recording

In order to assess central alpha and beta activity in sensorimotor areas we performed the following analyses:

1) To assess differences among observation conditions across the time course of the event related modulation, for every band range considered, we extracted the power from four consecutive 1-second time windows (3 seconds during observation, 1 second of resynchronization phase). We then compared the logarithms of the respective power value per condition divided by baseline in a 3x4x2 ANOVA (with three levels of Condition (Still, Zoom, Steadicam), 4 levels of Time Window (1^st^– 3^rd^ second of observation and 1^st^ second of grey screen) and two levels of Hemisphere (right vs. left)).

2) To control for effects in occipital electrodes, the ANOVAs described above were repeated for the occipital electrodes in the same frequency ranges. Statistics were done using STATISTICA software and reported results were adjusted for multiple comparisons using Tukey Post hoc tests; all error bars in graphs represent standard errors.

As a mean of normalization, all data analyzed were log transformed first. After this transform, all data showed normal distribution according to Kolmogorov-Smirnov test as well as Lilliefors corrected Kolmogorov-Smirnov test. In case of violation of sphericity, the data was Greenhouse Geisser corrected.

## Results

### EEG

#### Alpha bands: 8–14 Hz

**Fig 4:**
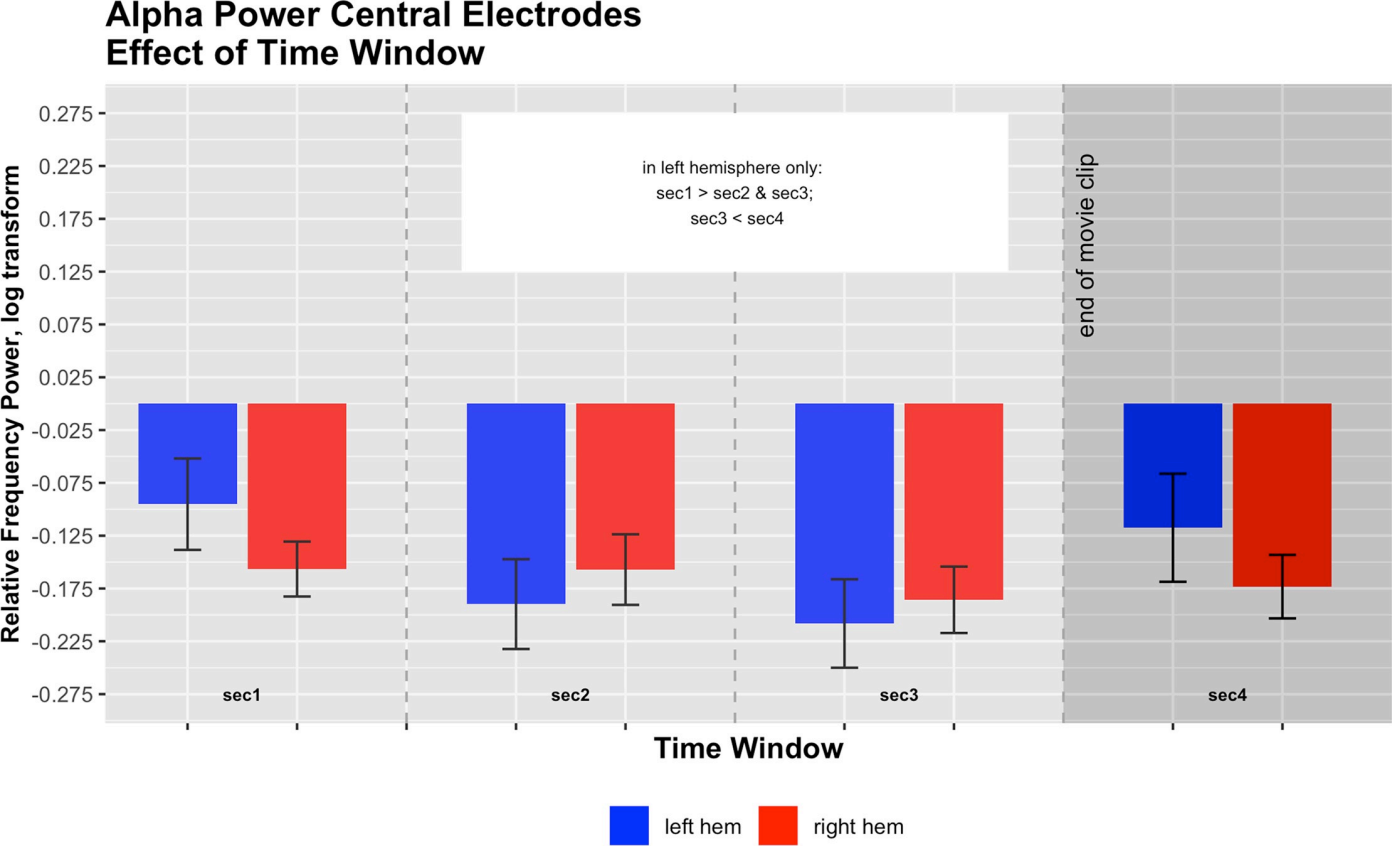
Central alpha frequency power (log corrected for normality) in the two hemispheres across the four time windows. Interaction effect F(2,3) = 4.21, p<0.05, partial eta squared = 0.22.

The 3x4x2 ANOVA (with three levels of Condition (Still, Zoom, Steadicam), 4 levels of Time Window (1^st^– 3^rd^ second of observation and 1^st^ second of grey screen) and two levels of Hemisphere (right vs. left)) for the selected alpha frequency ranges in central electrodes revealed a significant interaction of Hemisphere x Time Window only (F(2,3) = 4.21, p<0.05, partial eta squared = 0.22). Descriptives showed that frequency power was more strongly modulated in the left hemisphere, resulting in a typical ERD-ERS-rebound pattern (see also Avanzini et al., 2012). Left hemisphere: 1^st^ second M = -0.1 SE = 0.07; 2^nd^ second M = -0.19, SE = 0.07; 3^rd^ second M = -0.21, SE = 0.07; 4th second M = -0.12, SE = 0.08; Right hemisphere 1^st^ second M = -0.16 SE = 0.04; 2^nd^ second M = -0.16, SE = 0.05; 3^rd^ second M = -0.19, SE = 0.05; 4th second M = -0.17, SE = 0.04. Post-hoc supported this lateralization by showing significant differences between first and second (p<0.01) and first and third time window (p<0.001) (both desynchronization), as well as between third and fourth time window (p<0.05) (resynchronization) in the left hemisphere only. For illustration, see Fig 4.

We suggest that the hemispheric difference found may be due to a general dominance of the left hemisphere with regard to motor activations, causing the ERD-ERS pattern to be only detected here. We furthermore suggest that the detection of a typical ERD-ERS pattern during the observation of our stimuli that involve camera movements essentially supports the hypothesis that even if there is no actor in the clips, observing videos including those movements alone can lead to a modulation of central alpha frequency bands, likely reflecting sensorimotor activation. However, the lack of any significant main effect or interaction involving conditions, means that results of alpha frequency bands do not support the additional hypothesis that the different camera movement conditions elicit different sensorimotor-activations.

The same analysis in occipital electrodes showed a significant main effect of Condition (F(2,30), p<0.05, partial eta squared = 0.18) and of Time Window (F(3,45) = 6.75, p<0.001, partial eta squared = 0.31). Descriptives regarding the Condition effect can be summarized as follows: Still M = -0.28, SE = 0.06; Zoom M = -0.32, SE = 0.08; Steadicam M = -0.34, SE = 0.07. Post-hoc showed that frequency power for Still condition was significantly higher than for Steadicam condition (p<0.05).

Descriptives regarding the Time Window effect can be summarized as follows: 1^st^ second M = -0.22, SE = 0.07; 2^nd^ second M = -0.37, SE = 0.08; 3^rd^ second: M = -0.37, SE = 0.08, 4^th^ second: M = -0.29, SE = 0.07. Post-hoc showed that frequency power in the first time window was significantly higher than in the second and the third time windows (p<0.01 for both).

We suggest that these effects mirror attention effects as expected: occipital alpha rhythms are known to desynchronize in response to exposure to a visual stimulus without hemispheric differences. Furthermore, the strength of this desynchronization is known to be positively correlated to the amount of visual change involved, that indeed was strongest for the Steadicam condition.

#### Low beta bands: 14-20Hz

The 3x4x2 ANOVA for the selected low beta frequency range in central electrodes revealed a main effect of Condition (F(2,30) = 9.67, p<0.001, partial eta squared = 0.39), a main effect of Time Window (F(1.53,23.01) = 5.95, p<0.05, partial eta squared = 0.28), and a significant interaction of Condition x Time Window (F(6,90) = 5.52, p<0.001, partial eta squared = 0.27). Descriptives regarding the Condition effect can be summarized as follows: Still M = -0.07, SE = 0.02; Zoom M = -0.11, SE = 0.03; Steadicam M = -0.12, SE = 0.03. Post-hoc showed that frequency power for Still condition was significantly higher than for Zoom condition (p<0.01) and for Steadicam condition (p<0.001).

Descriptives regarding the Time Window effect can be summarized as follows: 1^st^ second M = -0.06, SE = 0.03; 2^nd^ second M = -0.13, SE = 0.03; 3^rd^ second: M = -0.15, SE = 0.03, 4^th^ second: M = -0.05, SE = 0.03. Post-hoc showed that frequency power in the first time window was significantly higher than in the third time window (p<0.05), and frequency power in the second and third time windows were significantly lower than in the fourth time window.

Descriptives regarding the Condition x Time Window interaction can be summarized as follows:

1^st^ second: Still M = -0.06, SE = 0.03; Zoom M = -0.08, SE = 0.04; Steadicam M = -0.05, SE = 0.03; 2^nd^ second: Still M = -0.06, SE = 0.03; Zoom M = -0.12, SE = 0.03; Steadicam M = -0.21, SE = 0.04;

3^rd^ second: Still M = -0.1, SE = 0.03; Zoom M = -0.16, SE = 0.05; Steadicam M = -0.19, SE = 0.03;

4^th^ second: Still M = -0.04, SE = 0.03; Zoom M = -0.07, SE = 0.04; Steadicam M = -0.03, SE = 0.04.

**Fig 5:**
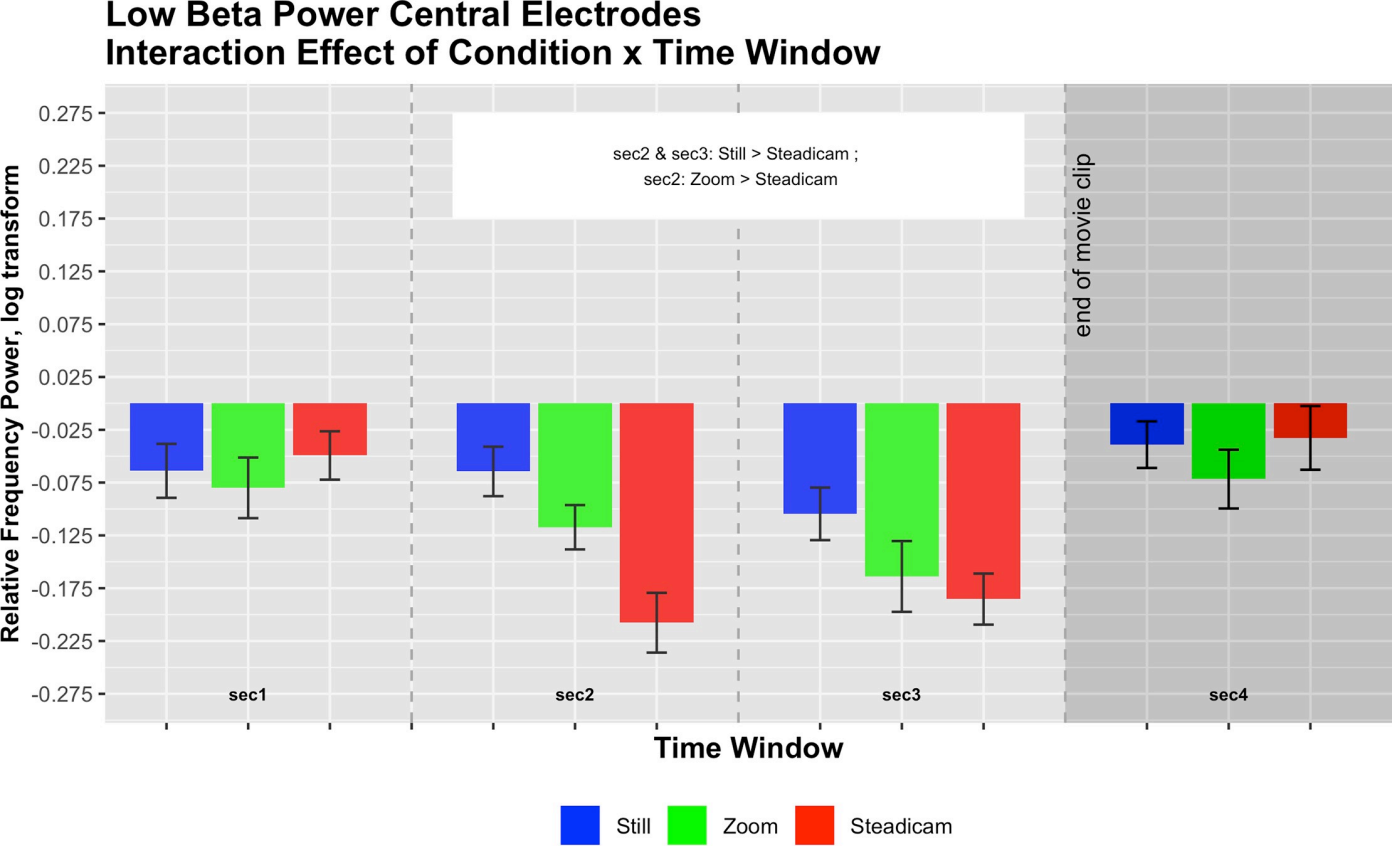
Central low beta frequency power (log corrected for normality) in the three observation conditions across the four time windows. Interaction effect F(6,90) = 5.52, p<0.001, partial eta squared = 0.27.

Post-hoc revealed that in the 2^nd^ second frequency power for Still condition and for Zoom condition was significantly higher than for Steadicam condition (p<0.001 for comparison with Still, p<0.05 for comparison with Zoom). In the 3^rd^ second frequency power for Still condition was significantly higher than for Steadicam condition (p<0.05). For illustration, see Fig 5.

We suggest that the main effect for Time Window reflects differences caused by a typical ERD and ERS pattern of central low beta band activity, representing the activation of the sensorimotor cortex during video observation. These results indicate that even if there is no actor in the clips, observing videos involving camera movements alone can lead to sensorimotor activation. We furthermore propose that the Condition and the Condition x Time effects reflect a modulation of the sensorimotor activation elicited by the different camera movement conditions. In particular, post-hocs of the Condition x Time effect in the second time window (reflecting desynchronization) show that this activation, thus the ERD, is significantly lower for the Still and Zoom conditions in comparison with the Steadicam condition. We take this as supporting our second initial hypothesis, positing that the Steadicam elicits the strongest activation of the central areas.

The same analysis in occipital electrodes showed a main effect of Condition (F(1.44,21.57) = 4.48, p<0.05, partial eta squared = 0.23), a main effect of Time Window (F(3,45) = 20.54, p<0.001, partial eta squared = 0.58), and a significant interaction of Condition x Time Window (F(6,90) = 6.46, p<0.001, partial eta squared = 0.3). Descriptives regarding the Condition effect can be summarized as follows: Still M = -0.13, SE = 0.04; Zoom M = -0.17, SE = 0.05; Steadicam M = -0.16, SE = 0.05. Post-hoc showed that frequency power for Still condition was significantly higher than for Zoom condition (p<0.05).

Descriptives regarding the Time Window effect can be summarized as follows: 1^st^ second M = -0.18, SE = 0.05; 2^nd^ second M = -0.19, SE = 0.05; 3^rd^ second: M = -0.19, SE = 0.05, 4^th^ second: M = -0.05, SE = 0.05. Post-hoc showed that frequency power in the first three time windows was significantly lower than in the fourth time window (p<0.001).

Descriptives regarding the Condition x Time Window interaction can be summarized as follows:

1^st^ second: Still M = -0.13, SE = 0.04; Zoom M = -0.23, SE = 0.06; Steadicam M = -0.17, SE = 0.05; 2^nd^ second: Still M = -0.15, SE = 0.05; Zoom M = -0.2, SE = 0.05; Steadicam M = -0.22, SE = 0.05;

3^rd^ second: Still M = -0.12, SE = 0.04; Zoom M = -0.2, SE = 0.05; Steadicam M = -0.24, SE = 0.05;

4^th^ second: Still M = -0.09, SE = 0.05; Zoom M = -0.05, SE = 0.05; Steadicam M = -0.02, SE = 0.06.

Post-hocs revealed that in the 1st second frequency power for Still condition was significantly higher than for Zoom condition (p<0.05). In the 3^rd^ second frequency power for Still condition was significantly higher than for Steadicam condition (p<0.01)

We suggest that the main Time Window effect mirrors a general attention effect as expected (desynchronization of occipital rhythms due to visual stimulus). This most likely also accounts for the difference between Still and Steadicam conditions in the 3^rd^ second (with Steadicam being the condition with the greatest visual changes). The results of the Condition x Time Window effect in the 1^st^ second and the main Condition effect require further exploration (see *discussion* section).

#### Middle beta bands: 18–24 Hz

The 3x4x2 ANOVA for the selected middle beta frequency range in central electrodes revealed a main effect of Condition (F(2,30) = 5.87, p<0.01, partial eta squared = 0.28), a main effect of Time Window (F(1.66,24.94) = 4.32, p<0.05, partial eta squared = 0.22), a significant interaction of Condition x Time Window (F(6,90) = 5.96, p<0.001, partial eta squared = 0.28) and a significant interaction of Condition x Hemisphere (F(2,30) = 7.59, p<0.01, partial eta squared = 0.34). Descriptives regarding the Condition effect can be summarized as follows: Still M = -0.05, SE = 0.03; Zoom M = -0.08, SE = 0.03; Steadicam M = -0.08, SE = 0.03. Post-hoc showed that frequency power for Still condition was significantly higher than for Zoom condition (p<0.05) and for Steadicam condition (p<0.01).

Descriptives regarding the Time Window effect can be summarized as follows: 1^st^ second M = -0.06, SE = 0.04; 2^nd^ second M = -0.08, SE = 0.03; 3^rd^ second: M = -0.1, SE = 0.02, 4^th^ second: M = -0.03, SE = 0.02. Post-hoc showed that frequency power in the third time window was significantly lower than in the fourth time window (p<0.01).

Descriptives regarding the Condition x Time Window interaction can be summarized as follows:

1^st^ second: Still M = -0.06, SE = 0.04; Zoom M = -0.08, SE = 0.04; Steadicam M = -0.05, SE = 0.04; 2^nd^ second: Still M = -0.04, SE = 0.03; Zoom M = -0.07, SE = 0.04; Steadicam M = -0.14, SE = 0.03;

3^rd^ second: Still M = -0.07, SE = 0.02; Zoom M = -0.11, SE = 0.03; Steadicam M = -0.12, SE = 0.03;

4^th^ second: Still M = -0.04, SE = 0.03; Zoom M = -0.04, SE = 0.02; Steadicam M = -0.01, SE = 0.03.

**Fig 6:**
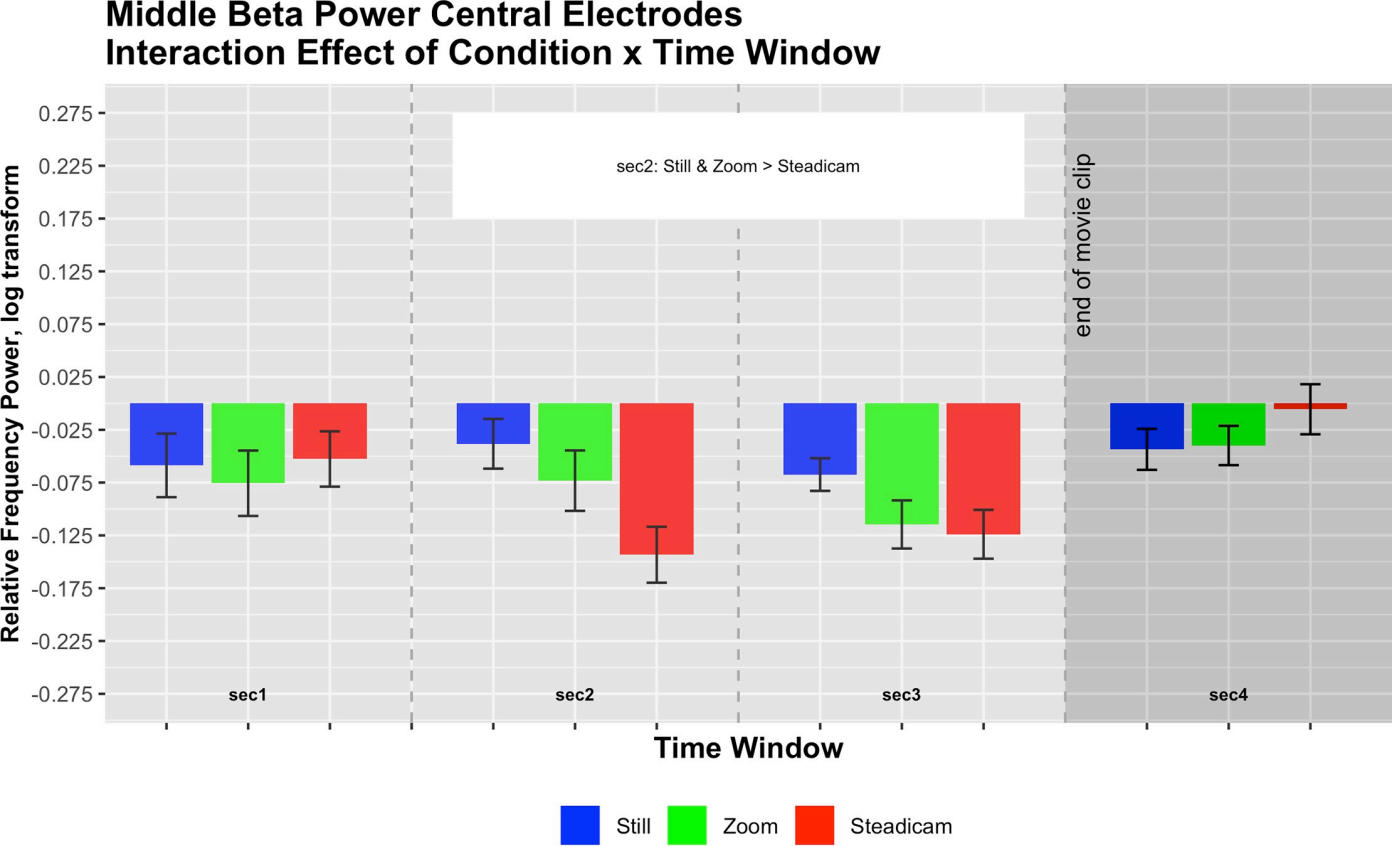
Central middle beta frequency power (log corrected for normality) in the three observation conditions across the four time windows. F(6,90) = 5.96, p<0.001, partial eta squared = 0.28.

Post-hoc revealed that in the 2^nd^ second frequency power for Still condition and for Zoom condition was significantly higher than for Steadicam condition (p<0.001 for comparison with Still, p<0.05 for comparison with Zoom). Please see for illustration Fig 6.

Descriptives regarding the Condition x Hemisphere interaction can be summarized as follows:

Left hemisphere: Still M = -0.04, SE = 0.02; Zoom M = -0.09, SE = 0.03; Steadicam M = -0.08, SE = 0.02; Right hemisphere: Still M = -0.06, SE = 0.03; Zoom M = -0.06, SE = 0.03; Steadicam M = -0.08, SE = 0.04.

Post-hoc revealed that frequency power for Still condition was higher than for Zoom and Steadicam only in the left hemisphere.

The main effect for Time Window reflects differences caused by a typical ERD and ERS pattern of central middle beta bands activity, representing the activation of the sensorimotor cortex during videos observation. Consequently, also these results indicate that even if there is no actor in the clips, observing videos involving camera movements alone can lead to a sensorimotor activation. Also here, the Condition, the Condition x Time and the Condition x Hemisphere effects reflect a modulation of the sensorimotor activation elicited by the different camera movement, especially in the left hemisphere. This hemispheric difference may be due to a general dominance of the left hemisphere with regard to motor activations, causing the ERD ERS pattern to be enhanced there. Furthermore, parallel to the results in low beta frequency bands, also in middle beta post-hoc of the Condition x Time effect in the second time window (reflecting desynchronization) showed that the sensorimotor activation was significantly lower for the Still and Zoom conditions in comparison with the Steadicam condition. Again, we take this as further supporting our second initial hypothesis, positing that the Steadicam elicits the strongest activation of the central areas.

The same analysis in occipital electrodes showed a main effect of Time Window (F(3,45) = 8.72, p<0.001, partial eta squared = 0.37), and a significant interaction of Condition x Time Window (F(6,90) = 4.66, p<0.001, partial eta squared = 0.24).

Descriptives regarding the main effect of Time Window can be summarized as follows: 1^st^ second M = -0.09, SE = 0.05; 2^nd^ second M = -0.12, SE = 0.05; 3^rd^ second: M = -0.13, SE = 0.04; 4^th^ second: M = -0.03, SE = 0.04. Post-hoc showed that frequency power in the second and the third second was significantly lower than in the fourth second (p<0.001).

Descriptives regarding the Condition x Time Window interaction can be summarized as follows:

1^st^ second: Still M = -0.08, SE = 0.05; Zoom M = -0.12, SE = 0.05; Steadicam M = -0.06, SE = 0.04; 2^nd^ second: Still M = -0.1, SE = 0.05; Zoom M = -0.12, SE = 0.05; Steadicam M = -0.15, SE = 0.05;

3^rd^ second: Still M = -0.1, SE = 0.04; Zoom M = -0.15, SE = 0.05; Steadicam M = -0.15, SE = 0.03;

4^th^ second: Still M = -0.05, SE = 0.04; Zoom M = -0.04, SE = 0.04; Steadicam M = 0.0, SE = 0.04.

Post-hoc revealed no significant differences between conditions in the single time windows, but only time course effects within conditions mirroring the main Time Window effect.

We suggest that such Time Window effect mirrors a general attention effect as expected (desynchronization of occipital rhythms due to visual stimulus).

### Interview results

In the post-EEG interview, we showed the participants the videos again (one for each Condition) and asked them whether they spotted or experienced any difference between the observed film clips, and if so, how they would describe them. We explicitly did not further specify the question, in order to not prime the participants. All participants reported to have seen three different types of clips, distinguished by the way of “filming” the scene. Participants further specified the two different modes of movement as illustrated by the following paradigmatic quotes (translated from the Italian) from three participants:

Participant 1: “The way the camera approaches the scene is different. In one (condition, added by authors), I see a zoom in the other a real movement. Actually, it looks as if a person is approaching the scene”.

Participant 3: “It looks like a person is looking at the table and sometimes approaching it. It looks more so for the videos in which the camera moves more.”

Participant 14: “I felt more involved when the camera was carried towards the table by a person, than when only a zoom was applied”

Interestingly, eight participants used their bodies to imitate the walking action, mostly holding a virtual camera in their hands, to illustrate the difference they saw among the videos.

Participants’ remarks as well as their body gestures during interviews showed that they were aware that the differences were caused by different ways of using the camera. While nobody explicitly mentioned a steadicam, all specifically referred to these videos as involving a “real” movement, often further specified as the involvement of a walking human holding the camera by hand. Some of them also reported that this gave them the feeling of seeing through the eyes of someone in the scene or walking themselves towards the table in the scene. Participants’ articulate statements show that they experienced the clips filmed with the steadicam as the most realistic ones and the best resembling a person’s movement approaching the scene.

## Discussion

In a previous study [19], we investigated whether the specific use of a camera or lens movement would modulate the spectators’ mirror mechanism when viewing a hand action performed by an actor on the screen. The results of that study showed that approaching the scene with a camera, and specifically with a steadicam, correlated with stronger ERD of the mu rhythm compared to watching the same scene filmed from a fixed distance. However, these results raised further questions regarding the precise nature of the motor simulation at stake. As a first possibility, they could indicate that the steadicam added ecological validity to the presentations, leading to stronger activation of the mirror mechanism in response to the observation of the hand actions executed by the actor in the scene. Indeed, previous studies indicated that the specific way of presenting an action is an important factor as, for example, a consistent number of mirror neurons in macaques respond more intensely to live actions than to videos of the same actions [38].

An alternative or additional explanation of the stronger sensorimotor ERD in our previous study on camera movements might refer to the walking action of the camera man itself. Indeed, previous research has shown that the sensorimotor ERD is sensitive to the observation of the outcomes of hand actions, such as the traces of hand movements captured in hand written letters and scribbles, brushstrokes and cuts on canvas [39, 40, 41]. Such explanation would also be congruent with the spectators’ and film-makers’ suggestion [20] that the steadicam lets spectators “embody” the camera, either by allowing them to take the position of a ‘quasi-character’ moving through the film scene [42] or at least by providing a link to the “marks” left by the cameraman.

The present study was set up to test this latter hypothesis by showing clips of a scene that did not include any acting human agent. We expected to find significant differences of the recorded sensorimotor ERD between Still condition compared with the two observation conditions that involved moving frames. The strongest ERD we expected to be elicited by the clips filmed by means of the steadicam. We expected this to correlate with interview results, indicating that clips filmed with the steadicam were experienced as best simulating a human movement through the scene.

To begin with, the results of our assessment of spectators’ subjective experience of the scenes confirmed our hypothesis, suggesting that clips produced with the Steadicam can create the strongest immersion and engagement with the filmed content.

The analysis of frequency power in central electrodes first of all indicated a typical ERD-ERS pattern in alpha, lower and middle beta frequencies for all camera conditions.

Occipital results in alpha bands diverged from these results clearly by not showing the same hemispheric difference as found in centrals. In beta bands, also occipital results showed a general ERD-ERS structure, though this is likely be due to attentional effects.

As no goal-related action was visible in the scene, the central results are thus likely due to the activation of the mirror mechanism by the observation of lens and/or camera movements.

Furthermore, in the lower beta bands and in the middle beta bands, the Steadicam condition showed a significantly stronger ERD than the Still and the Zoom conditions. No significant difference was found between Still and Zoom conditions.

Again, the single difference between Still and Steadicam found in low beta frequencies in occipital electrodes is likely reflecting an attention effect.

Taken together, this is evidence that the camera movement (Steadicam) is most effective in activating the sensorimotor cortex.

Notibly, these results are very close to what we found in our previous study[19], including the fact that strongest differences were found in the beta bands. It has been argued before that the beta component of the rolandic mu rhythm reflects motor activation more strongly than the alpha component [43]. Further research into the functional differences reflected in the different frequency bands is needed though [44, 45].

It is worth repeating that occipital electrodes were not clear of effects in this sample. Most results can easily be explained as attentional effects, but specifically in the low beta frequency range, some results remain puzzling. In particular, we do not find obvious reasons why the Zoom condition in general, and especially in the first second of the video observation, should cause stronger desynchronization of occipital low beta frequencies. Further studies have to be designed to better explore possible attentional correlates here.

Taken together, our results show that using camera movements to simulate a distance reduction between spectators and the observed scene can elicit the activation of the motor cortex, also when no goal-related action is portrayed in the scene. Our results additionally indicate that this effect (thus the ERD) is specific to clips filmed using a steadicam, also rated as best simulating the approach of a human observer towards the scene.

Establishing the discussed effect of camera and lens movement would have strong implications for our understanding of the motor and mirror neuron system in general, as well as for possible applications in, e.g., neuro-rehabilitation interventions or stimulus designs for future experiments. They could be also interesting in the research field of brain-computer interface because they demonstrate that beside the already documented motor imagery [46, 47], also the visual stimuli employed in our study were able to activate the sensorimotor EEG alpha and beta bands that can be used for the online control of external devices.

Insights on the relevance of embodied mechanisms for our engagement with moving images can inform film making and film theory and foster our understanding of the processes underlying our engagement with filmic scenes.

## Supporting information

S1 Fig Video clip of the Still condition (filmed with not moving camera mounted on tripod). (AVI)

S2 Fig (Example) of video clip of the Zoom condition (filmed via an automatic zoom (predetermined in speed and start as well as end frame)). (AVI)

S3 Fig (Example) of video clip of the Steadicam condition (filmed with a camera that was carried towards the table by a cameraman using a steadicam in such a way that the end frame matched that of the Zoom condition). (AVI)

S1 Tables Complete datasets for statistical analysis and figures, separated by frequency band and electrode location. (XLSX)
